# Comparison of Recurrence Patterns and Salvage Treatments After Definitive Radiotherapy for cT1a and cT1bN0M0 Esophageal Cancer

**DOI:** 10.3389/fonc.2022.857881

**Published:** 2022-07-11

**Authors:** Terufumi Kawamoto, Naoto Shikama, Shinji Mine, Keisuke Sasai

**Affiliations:** ^1^ Department of Radiation Oncology, Graduate School of Medicine, Juntendo University, Tokyo, Japan; ^2^ Department of Esophageal and Gastroenterological Surgery, Graduate School of Medicine, Juntendo University, Tokyo, Japan

**Keywords:** superficial esophageal cancer, chemoradiotherapy, salvage therapy, patterns of failure, carcinoma

## Abstract

**Background:**

Definitive radiotherapy (RT) for stage I esophageal cancer was reported to result in noninferior overall survival (OS) compared with surgery. However, only a few detailed reports of recurrence patterns and subsequent salvage treatments have been published. This study aimed to compare recurrence patterns and subsequent salvage treatments after definitive RT or chemoradiotherapy (CRT) between cT1a and cT1bN0M0 esophageal cancer (EC).

**Methods:**

Patients with cT1a or cT1bN0M0 esophageal squamous cell carcinoma who received definitive RT or CRT were included. Survival outcomes, recurrence patterns, and salvage treatments were evaluated.

**Results:**

In total, 40 patients with EC receiving RT or CRT were divided into two groups for evaluation: cT1a (20 patients) and cT1b (20 patients) groups. The 3-year OS rates were 83% and 65% (p = 0.06) and the 3-year progression-free survival rates were 68% and 44% (p = 0.15) in the cT1a and cT1b groups, respectively. Among those in the cT1a group, six had local recurrence and two had metachronous recurrence. Seven patients underwent salvage endoscopic submucosal dissection and one patient received argon plasma coagulation treatment. Among those in the cT1b group, six had local recurrence, one had regional recurrence, and one had both. Of these, one underwent salvage endoscopic submucosal dissection, one received photodynamic therapy, three underwent surgery, one received RT, and two received the best supportive care. Compared with the cT1b group, the cT1a group had a higher proportion of patients who underwent endoscopic treatments (p = 0.007). After the endoscopic treatments, no recurrences were observed in both groups.

**Conclusions:**

Regional recurrence and distant metastasis were not observed in the cT1a group. A higher proportion of patients in the cT1a group received salvage endoscopic treatments, and their OS tended to be favorable.

## Introduction

Esophageal cancer (EC) is the eighth most common cancer and the sixth leading cause of cancer-associated death globally (1). Owing to improvements in diagnostic measures, the number of patients diagnosedwith superficial EC has been increasing. According to the Comprehensive Registry of Esophageal Cancer in Japan, the incidence rate of clinical stage I cancer among all cancer cases increased from 23.1% in 1999 to 38.6% in 2013 (2).

Endoscopic resection is generally indicated for patients with tumors invading the cT1a-epithelium (EP)/lamina propria mucosa (LPM). For patients with tumors invading the cT1a-muscularis mucosa (MM), endoscopic resection or esophagectomy is the main treatment (3). However, in clinical practice, radiotherapy (RT) is often chosen as an alternative for patients with T1a EC depending on comorbidities, tumor localization, and extensive extension. For patients with tumors invading the cT1b-submucosa (SM), esophagectomy is the main treatment (3, 4). Recently, the outcomes of chemoradiotherapy (CRT) showed a noninferior trend compared with surgery in terms of overall survival (OS) in patients with cT1bN0M0 EC (5). However, elderly patients and those medically unsuitable for surgery were excluded or underrepresented in this trial, thus questioning the generalizability of the results for these populations. In recent years, favorable RT results have been reported for elderly patients and those medically unsuitable for surgery, including cT1a and cT1b EC (6–8). Moreover, only a few detailed reports discussed the patterns of recurrence and subsequent salvage treatments in these cases.Thus, this study aimed to compare the recurrence patterns and subsequent salvage treatments after definitive RT or CRT between cT1a and cT1b EC.

## Methods

### Study Population

This retrospective study protocol was reviewed and approved by the Juntendo Hospital review board (approval number: H20-0391). Informed consent was obtained *via* an opt-out method on the hospital’s website. This study was conducted in accordance with the Declaration of Helsinki.

We reviewed the medical records, RT treatment plans, and diagnostic images of patients with EC in the Juntendo Hospital between January 2009 and December 2020. Eligibility criteria were as follows: (i) presence of pathologically proven esophageal squamous cell carcinoma; (ii) presence of Eastern Cooperative Oncology Group performance status (ECOG PS) (9) scores of 0–2; (iii) presence of cT1a or cT1bN0M0 cancer based on the *UICC-TNM Classification, Eighth Edition* (10); and (iv) medically unsuitable for endoscopic resection and surgery or desire to receive RT. Patients who previously underwent endoscopic resection or other surgery and received RT or chemotherapy for EC were excluded. The same study population in T1a EC has been described previously (11). EC was diagnosed comprehensively based on the findings of upper gastrointestinal endoscopy, computed tomography (CT), and physical examination. Magnifying endoscopy and endoscopic ultrasonography were used for the clinical diagnostic differentiation of T1a-EP, LPM, T1a-MM, and T1b-SM1-3 EC (3). Comorbidities were estimated using the Charlson comorbidity index (CCI) based on 12 disease comorbidity categories (from 1 to 6 according to the relative risk of 1-year mortality) (12, 13). Any other active cancer was counted as two points.

### Treatment

External beam RT was administered using 6- or 10-MV X-rays of a linear accelerator. The daily fractional size of RT was 1.8–2.0 Gy based on the International Commission on Radiation Units and Measurements point; it was administered 5 days per week, with a total dose of 59.4–66 Gy. Either elective nodal irradiation (ENI), including the bilateral supraclavicular and mediastinal lymph node regions, or involved-field irradiation covering the primary tumor with a margin of 2–4 cm was used. Three-dimensional conformal RT was performed for all the patients. We used 2–4 fields to avoid the spinal cord. Among patients who received two-field irradiation, the beam direction was changed after irradiation with 40–41.4 Gy. ENI tended to be used in patients with normal respiratory and cardiac functions.

Chemotherapy was combined with RT in all patients except those with poor general conditions. The chemotherapy regimen consisted of either 5-fluorouracil (5 FU; 700 mg/m^2^ on days 1–4 every 4 weeks) plus cisplatin (CDDP; 70 mg/m^2^ on day 1 every 4 weeks) or docetaxel (DOC; 10 mg/m^2^ on day 1 per week). The 5-FU plus CDDP regimen tended to be used in patients with normal renal function, whereas DOC therapy tended to be used in older patients and those with deteriorating renal function. After treatment completion, the patients were followed up at 1- to 3-month intervals for the first 2 years and at 4- to 6-month intervals thereafter. Follow-up evaluations included history taking and physical examination, blood test, upper gastrointestinal endoscopy, and CT.

### Outcomes

The initial response was measured using the Response Evaluation Criteria in Solid Tumors guideline (version 1.1) (14) and based on endoscopy findings for the primary tumor according to the modified criteria of the 10th edition of the Japanese Classification of Esophageal Cancer established by the Japanese Society for Esophageal Disease. Complete response (CR) was defined as the disappearance of the primary tumor and the absence of irregular erosive, ulcerative, or elevated lesions as observed during endoscopy and/or the absence of malignant cells in biopsy specimens (15). Progressive disease (PD) was defined as distinct tumor growth or progression in esophageal stenosis compared with that at pretreatment. Incomplete response/stable disease (IR/SD) was defined as a response not meeting CR or PD. Radiological imaging studies, upper gastrointestinal endoscopy, and medical records of physical examinations were used to identify the recurrence sites. The presence of lesions outside the primary site was defined as metachronous recurrence, at the primary site was defined as local recurrence, and involvement of regional lymph nodes was defined as regional recurrence. Salvage treatments after the recurrence were also assessed. Toxicity was assessed and documented following the National Cancer Institute Common Terminology Criteria for Adverse Events version 5.0 (12, 15, 16). Toxicities were defined as acute and late if they occurred within and >3 months post-treatment, respectively.

### Statistical Analyses

The Mann–Whitney U test and Fisher’s exact test were used for assessing quantitative and qualitative data, respectively, and compare patient characteristics and toxicities between groups. OS, disease-specific survival (DSS), and progression-free survival (PFS) rates from the start of treatment were measured using the Kaplan–Meier method, and survival estimates were compared using the log-rank test. Death from any cause was defined as an event for calculating the OS rate, esophageal cancer-related death was defined as an event for calculating the DSS, and disease progression at any site or death from any cause was defined as an event for calculating PFS. All statistical analyses were performed using the EZR version 1.54 (17), and statistical significance was set at p < 0.05 (two-sided).

## Results

### Patients and Tumor Characteristics

Between January 2009 and December 2020, 75 patients with cT1a or cT1bN0M0 EC received definitive RT or CRT. Among them, 35 previously underwent endoscopic resection, and the remaining 20 in the cT1a and cT1b groups each received definitive RT or CRT as an alternative to endoscopic resection or surgery. The patient and tumor characteristics did not differ in patients between the two groups (**Table 1**).

**Table 1:** Patient and tumor characteristics.

Patient and tumor characteristics	cT1a group	cT1b group	p-value
Median age no.(range)	70 (41–82)	70 (52–86)	0.45
Sex no.(%)			0.13
Male	13 (65)	19 (95)	
Female	7 (35)	1 (5)	
ECOG PS no.(%)			0.75
0	6 (30)	10 (50)	
1	13 (65)	9 (45)	
2	1 (5)	1 (5)	
Location of primary tumor no.(%)			0.82
Cervix	1 (5)	3 (15)	
Upper thorax	0	1 (5)	
Middle thorax	15 (75)	13 (65)	
Lower thorax	3 (15)	2 (10)	
Abdomen	1 (5)	1 (5)	
Invasion depth no.(%)			
EP	0	–	
LPM	11 (55)	–	
MM	9 (45)	–	
SM1	–	8 (40)	
SM2	–	6 (30)	
SM3	–	6 (30)	
Median tumor craniocaudal length, cm (range)	6 (2–12)	6 (2–17)	0.88
Tumor craniocaudal length (cm)			1
< 5	5 (25)	6 (30)	
5–10	9 (45)	8 (40)	
≥ 10	6 (30)	6 (30)	
Tumor circumference no.(%)			0.055
< 1/3	0	2 (10)	
1/3–< 2/3	1 (5)	6 (30)	
2/3–< entire	4 (20)	2 (10)	
Entire	15 (75)	10 (50)	
Charlson comorbidity index no.(%)			0.42
0	5 (25)	8 (40)	
1	4 (20)	0	
2	6 (30)	7 (35)	
3	1 (5)	1 (5)	
4	3 (15)	3 (15)	
5	1 (5)	1 (5)	
Concurrent chemotherapy no.(%)			0.086
None	8 (40)	2 (10)	
DOC	11 (55)	14 (70)	
FP	1 (5)	4 (20)	
Total radiation dose no.(%)			0.49
59.4 Gy	0	1 (5)	
60 Gy	18 (90)	19 (95)	
66 Gy	2 (10)	0	
Radiation Field no.(%)			0.75
ENI	10 (50)	8 (40)	
IFI	10 (50)	12 (60)	

DOC, docetaxel; ECOG PS, Eastern Cooperative Oncology Group performance status; ENI, elective nodal irradiation; EP, epithelium; FP, 5-fluorouracil and cisplatin; IFI, involved-field irradiation; LPM, lamina propria mucosa; MM, muscularis mucosa; SM, submucosa.

The reasons for the patients’ unsuitability for endoscopic resection were tumor metastasis along the entire circumference of the tumor in 15 and 10 patients and widespread progression of the cancer in 6 and 6 patients (including duplicates) in the cT1a and cT1b groups, respectively. The reasons for patients’ unsuitability for surgery included comorbidities in 12 and 7 patients, double cancer in 5 and 7 patients, and desire to receive RT for esophageal conservation in 7 and 6 patients (including duplicates) in the cT1a and cT1b groups, respectively. The comorbidities were atrial fibrillation requiring anticoagulation in 6 and 2 patients, renal failure requiring dialysis in 4 and 0 patients, unstable angina requiring antiplatelet therapy in 2 and 3 patients, severe chronic obstructive pulmonary disease in 2 and 1 patients, chronic rheumatoid arthritis in 0 and 1 patients, hemophilia in 0 and 1 patients, and severe Parkinson’s disease in 1 and 1 patients (including duplicates) in the cT1a and cT1b groups, respectively. The median follow-up period was 67 (range, 13–131 months) and 29 (range, 13–83 months) for 14 and 11 survivors in the cT1a and cT1b groups, respectively. Among the 14 and 11 survivors in the cT1a and cT1b groups, 3 and 4 patients were lost to follow-up, respectively.

### Initial Response and Survivals

**Figure 1:**
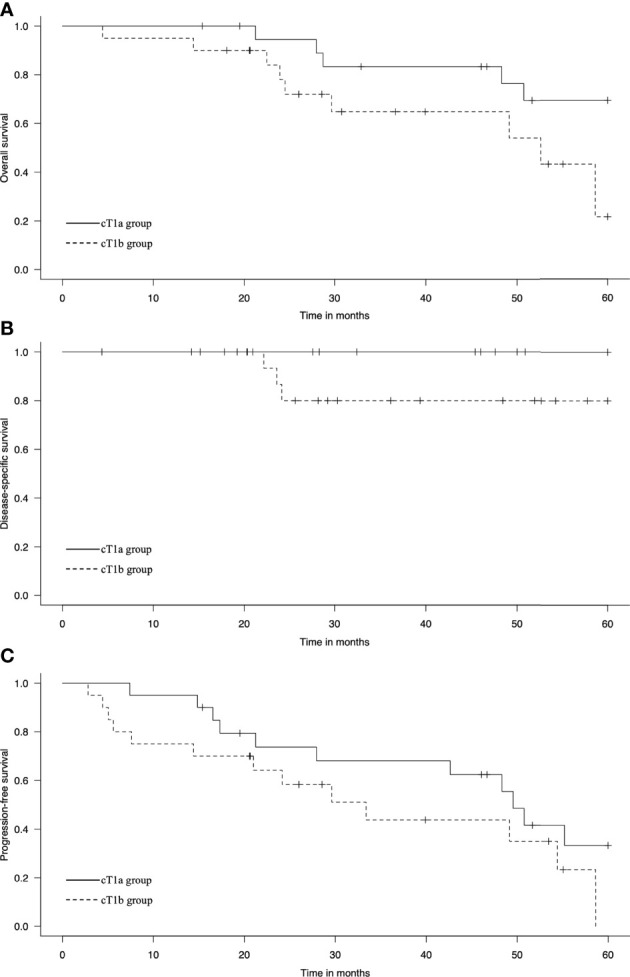
Kaplan–Meier estimates of **(A)** overall survival, **(B)** disease-specific survival, and **(C)** progression-free survival in the cT1a and cT1b groups.

At the initial treatment, 20 and 16 patients achieved CR in the cT1a and cT1b groups, respectively. Four patients achieved IR/SD in the cT1b group. The 3-year OS rates were 83% and 63% (p = 0.06), the 3-year DSS rates were 100% and 80% (p = 0.06), and the 3-year PFS rates were 68% and 44% (p = 0.15) in the cT1a and cT1b groups, respectively (**Figure 1**). Among the six patients in the cT1a group, three died of other cancers and the other three died of other causes, including chronic obstructive pulmonary disease (one patient) and aspiration pneumonia attributable to cerebral infarction (two patients). Out of nine patients in the cT1b group, three died of EC, two of other cancers, and four of other causes, including heart failure, radiation pneumonitis, bleeding after salvage surgery, and natural death due to unknown cause (one patient each).

### Toxicity

**Table 2** shows toxicities associated with RT or CRT. Grade 3 acute esophagitis was observed in 2 and 4 patients, grade 3 acute pneumonia in 1 and 0 patients, grade 3 white blood cell decrease in 1 and 1 patients, and grade 3 anemia in 0 and 2 patients in the cT1a and cT1b groups, respectively. Grade 4 esophagitis, grade 4 white blood cell decrease, grade 4 platelet count decrease, and grade 5 late pneumonitis were observed in 1 patient each in the T1b group.

**Table 2:** Treatment toxicities.

		cT1a group			cT1b group		
		Grade 1-2 no. (%)	Grade 3 no. (%)	Grade 4-5 no. (%)	Grade 1-2 no. (%)	Grade 3 no. (%)	Grade 4-5 no. (%)
Acute toxicity	Malaise	5 (25)	–	–	5 (25)	–	–
	Esophagitis	17 (85)	2 (10)	–	15 (75)	4 (20)	1 (5)
	Dermatitis	1 (5)	–	–	4 (20)	–	–
	Pneumonitis	–	1 (5)	–	1 (5)	–	–
	White blood cell decreased	9 (45)	1 (5)	–	8 (40)	1 (5)	1 (5)
	Anemia	4 (20)	–	–	9 (45)	2 (10)	–
	Platelet count decreased	4 (20)	–	–	9 (45)	–	1 (5)
Late toxicity	Dysphasia	2 (10)	–	–	1 (5)	–	–
	Pleural effusion	4 (20)	–	–	1 (5)	–	–
	Pericardial effusion	7 (35)	–	–	5 (25)	–	–
	Pneumonitis	3 (15)	–	–	3 (15)	–	1 (5)
	Hypothyroidism	3 (15)	–	–	–	–	–

### Recurrence Patterns and Salvage Treatments

**Table 3** summarizes the cases with recurrence. Recurrence occurred in eight patients each from both cT1a and cT1b groups. Among those in the cT1a group, six had local recurrence and two had metachronous recurrence. Metachronous recurrence was observed outside the radiation field in two patients. After identifying recurrence, seven patients underwent salvage endoscopic submucosal dissection (ESD), whereas one received argon plasma coagulation (APC). Among those in the cT1b group, six had local recurrence, one had regional recurrence, and one had both. Regional recurrence was observed outside (one patient) and within (one patient) the field of prophylactic irradiation. After identifying recurrence, among the patients with local recurrence, one underwent salvage ESD, one received photodynamic therapy (PDT), two underwent surgery for long craniocaudal tumor length and SM invasion, and two received the best supportive care for the onset of cerebral infarction and worsening hemophilia, respectively. Further, one patient with regional recurrence received RT and one with local and regional recurrence underwent surgery. Compared with the cT1b group, the cT1a group had a higher proportion of patients who underwent endoscopic treatments (p = 0.007). After endoscopic treatments, no recurrences were observed in both groups. After those in the cT1b group underwent salvage surgery, one patient died a month later owing to bleeding secondary to the surgery, one died 18 months later owing to liver metastasis, and one died 48 months later owing to heart failure, the original complication.

**Table 3:** Summary of recurrent cases.

Age	Sex	ECOG PS	Primay tumor location	Invasion depth	Tumor craniocaudal length (cm)	Tumor circumference	RT field	RT dose (Gy)	CRT	Months to disease recurrence	Recurrence lesions	Salvage therapy	Resected invasion depth	Resected tumor length (cm)	Tumor circumference	Sateus at last follow-up from salvage therapy
41	Male	0	Mt	MM	16	Entire	ENI	60	Yes	43	Local	ESD	EP	1.8	< 1/3	ANED 44 m
49	Female	0	Mt	MM	15	Entire	ENI	66	Yes	80	Local	ESD	EP	0.8	< 1/3	ANED 45 m
61	Male	1	Lt	LPM	10	Entire	IFI	60	Yes	15	Local	ESD	EP	1.4	< 1/3	ANED 104 m
62	Male	0	Mt	MM	3	Entire	ENI	60	Yes	17	Local	ESD	EP	0.5	< 1/3	ANED 73 m
65	Male	1	Ae	LPM	5	Entire	ENI	60	No	17	Local	ESD	EP	3	< 1/3	ANED 16 m
70	Male	1	Mt	MM	2	Entire	IFI	66	No	7	Metachronous	APC	–	–	< 1/3	DID 22 m
72	Male	1	Mt	MM	10	Entire	ENI	60	Yes	55	Local	ESD	EP	1.2	< 1/3	DID 7 m
73	Male	1	Mt	LPM	4	Entire	IFI	60	Yes	50	Metachronous	ESD	LPM	3.1	< 1/3	ANED 32 m
60	Male	0	Mt	SM2	5	Entire	IFI	60	Yes	33	Local	PDT	–	–	< 1/3	ANED 3 m
64	Male	0	Mt	SM3	6	Entire	ENI	60	Yes	54	Local	ESD	EP	0.9	< 1/3	ANED 26 m
66	Male	1	Ce	SM2	10	Entire	ENI	60	Yes	21	Locoregional	Surgery	Trachea	11	Entire	DE 1 m
66	Male	0	Mt	SM3	5	2/3	IFI	60	Yes	6	Regional	RT	–	–	–	DE 16 m
68	Male	2	Lt	SM2	6	Entire	IFI	60	Yes	5	Local	Surgery	SM2	7	Entire	DID 48 m
74	Female	1	Mt	SM2	5	Entire	ENI	60	Yes	3	Local	Surgery	SM1	7	2/3	DE 18 m
76	Male	1	Ce	SM1	8	Entire	IFI	60	Yes	24	Local	BSC	–	–	Entire	No follow-up
78	Male	1	Mt	SM1	17	Entire	IFI	59.4	Yes	8	Local	BSC	–	–	Entire	No follow-up

ANED, alive with no evidence of disease; APC, argon plasma coagulation; CRT, chemoradiotherapy; DID, died of intercurrent disease; DE, died of esophageal cancer; ECOG PS, Eastern Cooperative Oncology Group performance status; ENI, elective nodal irradiation; EP, epithelium; ESD, endoscopic submucosal dissection; IFI, involved-field irradiation; LPM, lamina propria mucosa; MM, muscularis mucosa; PDT, photodynamic therapy; RT, radiotherapy; SM, submucosa

## Discussion

The present study was designed to clarify differences in the recurrence patterns and subsequent salvage treatments of definitive RT or CRT between cT1a and cT1b EC. All patients in the cT1a group received salvage endoscopic treatments, whereas two patients in the cT1b group received salvage endoscopic treatments.

**Table 4** presents data of previous studies that examined the efficacy of RT for stage I EC (18–23). The local and metachronous recurrence rate in patients with cT1a EC (0%–29%) was relatively lower than that in patients with cT1b EC (23%–38%). The local and metachronous recurrence rate in our study was slightly high compared with the rates reported in previous studies. This might be associated with a longer tumor craniocaudal length in our study than that in previous studies. Previous studies reported that a long tumor craniocaudal length was a prognostic factor for local recurrence of superficial EC, consistent with our findings (19, 21). The regional recurrence and distant metastasis rates were 6%–12% and 1%–6% in those with cT1b EC, respectively, whereas neither of them were observed in those with cT1a EC, except in one previous study (22). The regional recurrence rate in our study was similar to the rates reported previously. A previous study reported regional metastasis rates of 0%, 9%–15%, and 41%–44% at the time of surgery among patients with EP/LPM, MM/SM1, and SM2/SM3 EC, respectively (24). In the cT1b group, the lower rate of regional recurrence after RT compared with that of regional metastasis at the time of surgery suggested that potential lymph node metastasis was suppressed by ENI and concurrent chemotherapy.

**Table 4:** Literature review of studies that included radiotherapy cases for cT1 aN0 M0 esophageal cancer.

Author	Year	no.	Median age (range)	Sex Male/Female (%)	PS 0/1-2 (%)	T Stage	Tumor craniocaudal length, cm (median [range])	Median prescribed dose (Gy)	ICBT (%)	CRT (%)	Field ENI/IFI (%)	OS rate (%)	Local and metachronous recurrence (%)	Reginal lymph node recurrence (%)	Distant metastassis (%)	Salvage treatments (%)	Salvage endscopic treatments (%)	Salvage surgery (%)
Nemoto	2001	52	68 (43–89)	85/15	NS	T1a	NS	65	63	3	0/100	62 (5y)	12	0	0	NS		
		95				T1b						42 (5y)	23	12	4			
Ishikawa	2006	18	70 (50–86)	89/11	39/61	T1a	Almost < 5 cm	60–70 (range)	33	0	100/0	100 (5y-DSS)	0	0	0	–	–	–
		50		76/24	36/64	T1b		60–73 (range)	60	0		75 (5y-DSS)	18	6	4	42	0	25
Yamada	2006	23	67 (48–83)	89/11	NS	T1a	3.6 (1–14)	59.4	83	100	100/0	85.2 (5y-DSS)	17	0	0	41	18	14
		40				T1b						70 (5y-DSS)	38	18				
Kodaira	2010	24	66 (41–89)	93/7	31/69	T1a	4 (1–16)	60	27	61	0/100	82 (3y)	26	6	1	56	41	8
		71				T1b												
Murakami	2012	44	70 (43–89)	92/8	79/21	T1a	Almost < 3 cm	54	100	0	100/0	84 (5y)	29	2	0	NS	45	
		43				T1b		60	100	0	100/0	31 (5y)	30	12	2			
Suzuki	2018	3	70 (59–87)	81/19	76/24	T1a	5 (1–20)	50	0	86	19/81	NS	0	0	0	–	–	–
		18				T1b						NS	28	6	6	71	29	0
Our report	2022	20	70 (41–82)	65/35	30/70	T1a	6 (2–16)	60	0	60	50/50	83 (3y)	40	0	0	100	100	0
		20	70 (52–86)	95/5	50/50	T1b	6 (2–17)	60	0	90	40/60	65 (3y)	35	10	0	75	25	38

DSS, disease-specific survival; ECOG PS, Eastern Cooperative Oncology Group performance status; ENI, elective nodal irradiation; ICBT, intracavitary brachytherapy; IFI, involved-field irradiation; NS, not stated; OS, overall survival.

*Including submucosal cancer.

When there is a local residual or recurrent lesion after definitive RT or CRT, salvage surgery or endoscopic treatment may allow long-term survival. In case of medically unsuitable for salvage surgery or endoscopic treatment, patients are indicated for chemotherapy or best supportive care (25). Previous studies reported that R0 resection allowed long-term survival in salvage surgery. However, salvage surgery increased the incidence of postoperative complications and in-hospital mortality (26, 27). When a residual lesion remained confined in the MM, salvage endoscopic treatment can be performed safely (28). Salvage PDT for lesions within the SM or muscularis propria showed a high local CR rate with acceptable safety after the failure of definitive CRT (29). However, in Japan, PDT could only be performed in a few facilities, which may be the reason why the rate of salvage endoscopic treatments was low.

In our study, all patients with cT1a EC who were unsuitable for endoscopic resection as an initial treatment because of cancer metastasis along the entire circumference or a wide extent of tumor involvement could be treated with salvage ESD or APC. This can be attributed to the effect of regular follow-up with endoscopy. A previous study reported that cT1-2 and N0 stage cancers at baseline treated with salvage endoscopic resection were significant factors of good prognosis in terms of OS (30). It should be noted that local recurrence was observed in the one case more than 7 years after CRT. Thus, long regular follow-up with endoscopy and multidisciplinary treatment was considered important for the management of cT1a EC.

Among patients with cT1b EC with recurrence, <50% (including our study) could receive salvage endoscopic treatments (19–23). Local recurrences in the cT1a group had a shorter craniocaudal tumor length than the original tumor and could be treated endoscopically, whereas three patients in the cT1b group had a longer craniocaudal tumor length than the original tumor and required surgery. T1b EC may have a faster tumor growth rate than T1a EC. In the cT1b group, the invasion depth of local recurrence was deeper than the SM, except in one patient in our study. It should be noted that local and regional recurrence was observed in most cases within 2 years after CRT. Thus, frequent regular follow-up with endoscopy and CT was considered important for the management of cT1b EC compared with cT1a EC, at least within 2 years. In our study, one of three patients who underwent salvage surgery died of bleeding. In contrast, a recent study reported that salvage surgery was relatively safe (31). Among 96 patients who received RT with a total dose of 50.4 Gy, 25 underwent salvage surgery, with a 3-year survival rate of 48%. In their cohort, pulmonary complications, suture failure, and treatment-related death were observed in 4%, 12%, and 4% of patients, respectively. Nevertheless, salvage surgery after high-dose irradiation was considered to result in more complications and treatment-related deaths than conventional esophagectomy or salvage surgery after RT with a total dose of 50.4 Gy. Considering the outcomes of salvage surgery, RT with a total dose of 50.4 Gy might be an appropriate treatment for stage I EC. To establish a new treatment option, Japanese study groups are conducting a phase III clinical trial comparing CRT with a dose of 50.4 and 60 Gy for treating cT1bN0M0 EC [Japan Registry of Clinical Trials (jRCT) study number: jRCTs031200067].

The present study has several limitations associated with its retrospective design. First, the sample size was small, which affects the statistical power. Second, the external validity might be low. Some institutions performed subtotal-to-total circumferential resection with prophylactic steroids for more than three-fourths of the circumference of the EC (32, 33). A phase III study aimed at prospectively evaluating the stenosis-preventive effect of submucosal triamcinolone injection and oral prednisolone treatment is ongoing (34). However, RT may be necessary for patients at a high risk for esophageal stricture despite treatment with prophylactic steroids.

In conclusion, regional recurrence and distant metastasis were not observed among patients in the cT1a group, whereas regional recurrence was observed among patients in the cT1b group after definitive RT or CRT. A higher proportion of patients in the cT1a group were able to receive salvage endoscopic treatments and their OS tended to be favorable compared with those in the cT1b group. Frequent regular follow-up with endoscopy and CT was considered important for the management of cT1b EC compared with cT1a EC, at least within 2 years.

## Data Availability Statement

The raw data supporting the conclusions of this article will be made available by the authors, without undue reservation.

## Ethics Statement

The studies involving human participants were reviewed and approved by Juntendo Hospital review board. Written informed consent for participation was not required for this study in accordance with the national legislation and the institutional requirements.

## Author Contributions

TK prepared the manuscript and performed the literature search. TK reviewed and edited the manuscript. TK, NS, SM, and KS reviewed the manuscript. All authors contributed to the article and approved the submitted version.

## Conflict of Interest

The authors declare that the research was conducted in the absence of any commercial or financial relationships that could be construed as a potential conflict of interest.

## Publisher’s Note

All claims expressed in this article are solely those of the authors and do not necessarily represent those of their affiliated organizations, or those of the publisher, the editors and the reviewers. Any product that may be evaluated in this article, or claim that may be made by its manufacturer, is not guaranteed or endorsed by the publisher.
